# Could ergothioneine serve as an indicator of functional plant-AMF interactions in crop plants?

**DOI:** 10.3389/fpls.2026.1894503

**Published:** 2026-09-28

**Authors:** Valter Weijola, Lena F. Nielsen, Benjamin Fuchs

**Affiliations:** 1 Department of Biodiversity Sciences, University of Turku, Turku, Finland; 2 Department of Agroecology, Aarhus University, Slagelse, Denmark

**Keywords:** arbuscular myccorhizal fungi (AMF), crop genotype, ergothioneine, nutrient exchange, plant microbe interactions

## Abstract

Improving the functional benefits of arbuscular mycorrhizal fungi (AMF) is considered an important part of developing more sustainable and resilient crop production systems. However, the outcomes of AMF symbioses vary substantially depending on environmental conditions, soil fertility, fungal community composition, and host genotype. Even among varieties of the same crop species, responses to AMF colonization can differ considerably, yet methods for quantifying functional plant–AMF interactions remain labour-intensive and often fail to capture symbiotic efficiency directly. Here, we propose that ergothioneine (EGT), a sulfur-containing antioxidant amino acid whose accumulation in plants appears to be strongly influenced by AMF-associated pathways, may serve as an integrated biochemical indicator of functional AMF-mediated nutrient exchange. We hypothesize that variation in plant EGT accumulation between crop varieties reflects differences in the regulation and functional performance of plant–AMF interactions, possibly including symbiotic signalling, carbon allocation, nutrient exchange efficiency, and/or rhizosphere microbial dynamics. We present new results showing differences in grain EGT concentration among five spring wheat varieties, supporting the possibility that host genotype influences AMF-associated EGT accumulation. We discuss potential mechanisms underlying these differences, evaluate alternative explanations, and outline experimental approaches for testing the relationship between EGT accumulation and functional plant–AMF interactions. If validated, EGT quantification could provide a scalable and straightforward approach for identifying crop genotypes with enhanced responsiveness to AMF-mediated nutrient acquisition, with potential applications in breeding and management strategies for low-input and ecologically sustainable agriculture.

## Introduction

Improving nutrient-use efficiency in crop production is an increasingly important challenge for global agriculture. In developing countries and under organic cropping systems, crop productivity is often constrained by limited nutrient availability, particularly phosphorus, while conventional fertilization practices contribute to environmental degradation, greenhouse gas emissions, and depletion of finite mineral resources (Lynch, 2007; Conijn et al., 2018; Bindraban et al., 2020; Carkner et al., 2023). Developing crop varieties capable of maintaining productivity under reduced fertilizer inputs has therefore become a major objective of sustainable agriculture (Veneklaas et al., 2012; Heuer et al., 2017).

Among biological interactions influencing nutrient acquisition, arbuscular mycorrhizal fungi (AMF) play a particularly important role. Most major crop species form symbiotic associations with AMF, whose hyphal networks extend beyond the root depletion zone and facilitate the acquisition of phosphorus, nitrogen, micronutrients, and water in exchange for plant-derived carbon compounds (Jakobsen et al., 1992; Smith and Read, 2008; Keymer et al., 2017). In addition to their role in nutrient acquisition, AMF interactions have also been associated with increased tolerance to drought, pathogens, and other abiotic stresses (Wehner et al., 2010; Mustafa et al., 2017; Mathur et al., 2019; Spagnoletti et al., 2021; Huang et al., 2023).

AMF additionally contribute to soil structure and ecosystem functioning through the production of glomalin-related soil proteins and through interactions with broader rhizosphere microbial communities (Wright and Upadhyaya, 1998; Driver et al., 2005; Zhang et al., 2022). These processes influence soil aggregation, microbial activity, carbon sequestration, and nutrient cycling (van der Heijden et al., 2015; Begum et al., 2019). Consequently, improving beneficial plant–AMF interactions can be considered a key component of resilient low-input agricultural systems (van der Heijden et al., 2008; Smith and Smith, 2011; Thirkell et al., 2017).

Despite their importance, functional plant–AMF interactions remain difficult to quantify. Traditional assessments often rely on microscopy-based measurements of root colonization intensity. However, colonization alone may correlate poorly with the actual nutritional benefits received by the host plant, as AMF function can vary widely depending on environmental context, host genotype and host–fungal combinations (Hetrick et al., 1993; Johnson et al., 1997; Smith and Smith, 2011). More direct methods for evaluating nutrient transfer, such as isotopic tracing experiments in compartmentalized systems (Pearson and Jakobsen, 1993; Ravnskov and Jakobsen, 1995), provide more accurate functional measures but are labor-intensive and require specialized infrastructure (Smith and Read, 2008). As a result, scalable indicators capable of reflecting functional AMF activity in crops remain limited.

Chemical markers of AMF association have been developed, including AMF-associated fatty acid signatures and plant-derived blumenol derivatives, which can provide proxies for fungal biomass or root colonization. However, these markers primarily indicate the presence or extent of AMF association rather than the nutritional benefits delivered to the host (van Aarle and Olsson, 2003; Wang et al., 2018; Olsson and Lekberg, 2022).

Ergothioneine (EGT) is a sulfur-containing amino acid with strong antioxidant properties that is synthesized by fungi and certain bacteria but not by plants or animals (Borodina et al., 2020). While EGT was discovered and characterized in 1909 and 1911 respectively (Tanret, 1909; Barger and Ewins, 1911), its potential biological importance was only understood in 2005 when it was discovered that humans and other vertebrates possess a EGT specific transporter protein (Gründemann et al., 2005). Since then, the number of studies on EGT and its effects on human health have increased dramatically. In humans, EGT has been linked to reduced risk of neurodegenerative, cardiovascular, and renal diseases, several cancers, and lower all-cause mortality (Cheah et al., 2016; Halliwell et al., 2018; Smith et al., 2020; Ba et al., 2021; Tian et al., 2023). Because plants do not synthesize EGT themselves, variation in its concentration in food crops depends on processes governing its acquisition and accumulation. Understanding these processes is therefore relevant both to crop nutritional quality and to the question of whether differences in plant–AMF interactions contribute to variation in EGT accumulation.

Recent studies suggest that AMF symbionts are important contributors to EGT accumulation in plants and that agricultural practices influencing soil microbial communities can substantially alter EGT concentrations in crops. For example, reduced tillage intensity and management systems that minimize disruption of AMF hyphal networks have been associated with increased EGT accumulation in wheat and other crops (Beelman et al., 2022; Carrara et al., 2023, 2024). Carrara et al. (2023) proposed that AMF may function as transport mediators for soil-derived EGT, or influence plant physiology related to EGT uptake, rather than in EGT synthesis. These findings support that plant EGT concentration reflects processes associated with active plant–fungal symbiotic interactions but the precise uptake mechanisms remains unresolved.

Although current evidence show a relationship between EGT accumulation and plant-AMF interactions, no attention has been given to the possibility that crop genotype itself may influence EGT accumulation. Such variation could result from multiple interacting mechanisms, including differences in host regulation of AMF symbiosis, and plant EGT transport- or storage physiology.

Building on novel data showing significant differences in grain EGT concentration among five spring wheat varieties (**Figure 1**), we consider the possibility that plant EGT accumulation may serve as an integrated indicator of functional plant–AMF interactions. Specifically, we hypothesize that variation in plant EGT concentration may reflect differences in AMF-associated nutrient exchange activity that are not captured by fungal root colonization alone, and could therefore provide a comparatively simple and scalable approach for assessing variation in plant responsiveness to AMF symbiosis. For EGT to fulfil this potential, its relationship with AMF activity would need to be consistent across environments and sensitive to biologically meaningful differences in symbiotic performance.

**Figure 1:**
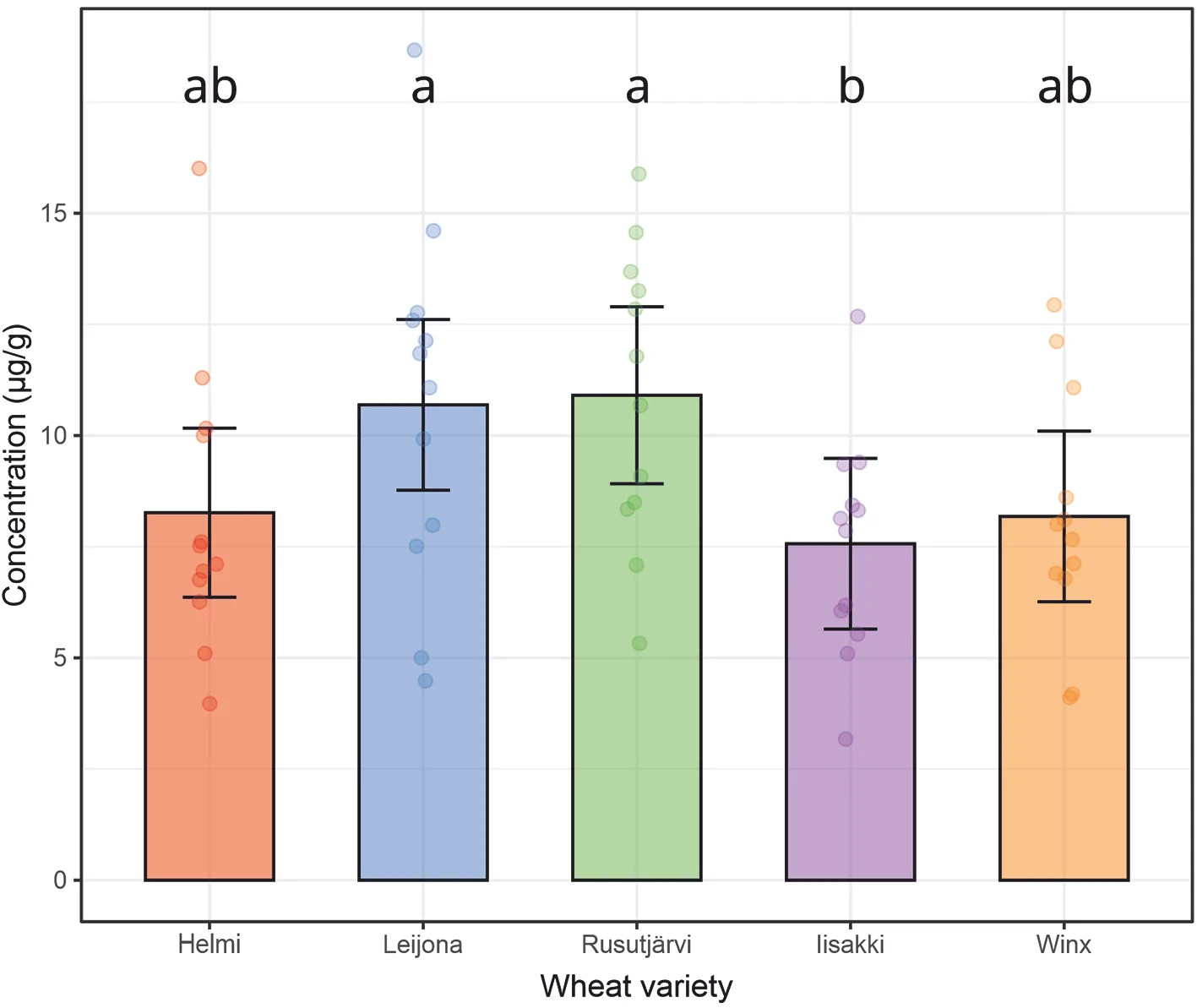
Grain EGT concentration among five spring wheat varieties. Points represent raw data, bars indicate estimated marginal means, and error bars show 95% confidence intervals. Different letters indicate significant differences among wheat varieties.

## Methods

### Field setup

The field experiment was conducted in southwest Finland (60°26′N, 22°10′E) at the University of Turku Botanical Garden in Ruissalo. Twelve cultivation stripes (1 × 25 m) were prepared using a reciprocating spader and rotary tiller to a depth of 15 cm. Five spring wheat varieties, including four modern cultivars commonly grown in Finland (Helmi, Winx, Leijona, and Iisakki) and one Finnish landrace (Rusutjärvi), were sown on May 15^th^ 2025 in individual 1 × 1 m plots within each stripe. Soil analyses from the previous year showed plant-available phosphorus concentrations of 8.1–11.5 kg P ha^-^¹ across plots, above the target range of 4.6–7.7 kg P ha^-^¹. Soil organic matter content ranged from 2.6 to 4.7%. The previous crop (2024) was also spring wheat. No fertilizers were used during the 2025 growing season.

At maturity (August 20^th^ to September 2^nd^), fifteen seed heads were collected from each plot, air-dried, and threshed, resulting in twelve replicate samples per variety. Grain samples were homogenized using a laboratory grinder, spiked with the internal standard L-(+)-ergothioneine-d9, and extracted using 70% methanol (MeOH). Extracts were sonicated and centrifuged, and the resulting supernatants were analyzed using liquid chromatography–mass spectrometry (LC–MS) based on the method described by Beelman et al. (2021), with modifications to suit available instrumentation and chromatographic conditions.

### Statistical analyses

The statistical analysis were completed in R version v. 4.3.2, using linear mixed-effects models (LMER). Stripe and row were included as random effects to account for the spatial layout of the field. The model assumptions of homoscedasticity and residual normality were assessed using residuals versus fitted plots and Q-Q plots, respectively. *Post hoc* pairwise comparisons among wheat varieties were performed using Tukey-adjusted estimated marginal means (emmeans package). Effect size was calculated using the effectsize package in R.

## Results

### Wheat ergothioneine concentration

Analysis revealed significant differences in EGT concentration among the five wheat varieties (F_4,35.95_ = 4.17, p = 0.0071, partial η² = 0.32). Mean EGT concentrations ranged from 7.6 to 10.9 µg g^-^¹ (**Figure 1**), with the Finnish landrace Rusutjärvi showing the highest mean concentration. Pairwise *post hoc* comparisons showed that Rusutjärvi and Leijona had significantly higher concentrations than Iisakki. These results demonstrate previously uncharacterized variation in grain EGT concentration among wheat varieties cultivated under shared field conditions. No measurements of AMF colonization or other indicators of AMF functionality were however included in the experiment.

## Discussion

Our observations revealed substantial differences in grain EGT concentration among five spring wheat varieties cultivated under shared field conditions, with the Finnish landrace Rusutjärvi exhibiting the highest mean EGT concentration. Although these observations do not establish causality, taken together with previous evidence linking AMF to EGT accumulation in plants (Carrara et al., 2023), they raise the possibility that variation in EGT accumulation among cultivars may partly reflect genotypic differences in plant–AMF interaction dynamics. Ergothioneine concentrations may be a particularly informative tool in this context because, unlike mineral nutrients such as phosphorus, zinc, or iron, which can be acquired both directly by the plant and through AMF-mediated pathways, plants do not synthesize EGT themselves and current evidence suggests that its accumulation is to some extent linked to AMF-associated uptake processes.

Variation in plant EGT accumulation is likely driven by multiple interacting processes associated with plant–AMF symbiosis as well as by plant physiological processes unrelated to symbiotic performance. We therefore propose that EGT concentration represents an integrated biochemical signal shaped by host-regulated symbiotic activity, host physiology, and environmental context.

Here, we focus primarily on the symbiotic component, as it may provide a useful proxy for identifying crop cultivars with enhanced responsiveness to AMF and improved adaptation to low-input production systems. Testing this hypothesis will require targeted experimental studies correlating EGT quantification with independent measurements of AMF function.

### Host symbiotic regulation and nutrient exchange efficiency

The potential mechanism with practical implications underlying genotype-dependent EGT accumulation involves differences in host regulation of AMF symbiosis. Host genotypes differ substantially in the chemical signals released into the rhizosphere, including sugars, flavonoids, strigolactones, and organic acids (Bais et al., 2006; Badri and Vivanco, 2009). These compounds influence AMF recruitment, colonization dynamics, and fungal metabolism (Akiyama et al., 2005; Gutjahr and Parniske, 2013).

Variation in root exudate composition may therefore influence both the establishment and functional activity of AMF networks associated with different crop varieties. Some varieties may support larger or more metabolically active fungal networks, resulting in greater nutrient exchange and potentially greater EGT transfer to the host plant.

Plant varieties could also differ in their regulation of arbuscule formation and maintenance, processes that are under strong host genetic control (Gutjahr and Parniske, 2013; Smith and Smith, 2011). Because arbuscules represent the primary site of nutrient exchange between AMF and host roots, variation in arbuscule abundance or persistence may influence the efficiency of nutrient transfer (Smith and Read, 2008). Genotypes allocating greater carbon resources to fungal partners may enhance reciprocal nutrient (including EGT) acquisition from AMF networks.

### Plant transport and tissue allocation

Variation in EGT accumulation may also arise from genotype-dependent differences in plant transport, storage, and metabolism. Crop genotypes may differ in root architecture, EGT uptake efficiency, vascular transport, tissue partitioning, and retention of EGT in leaves and developing grains. Differences in sulfur metabolism could further influence EGT accumulation independently of AMF activity. Although dedicated EGT transport systems have not yet been identified in plants, variation in amino acid transport pathways may influence uptake and intracellular allocation of EGT-like compounds (Tegeder and Ward, 2012; Rentsch et al., 2007). In addition, genotypic differences in phloem loading and grain filling processes can substantially alter the allocation of metabolites to developing seeds, independently of fungal activity (Patrick and Offler, 2001; Sreenivasulu and Wobus, 2013). Together with the fact that EGT accumulation depends on uptake rather than endogenous synthesis in plants (Borodina et al., 2020), these mechanisms provide plausible alternative or (perhaps more likely) additional explanations for variation in grain EGT concentration.

### Rhizosphere microbial interactions and environmental context

Environmental conditions are also likely to influence the relationship between EGT accumulation and plant–AMF interactions. Soil phosphorus availability, moisture conditions, rhizosphere bacterial communities, tillage intensity, and broader microbial disturbance are all known to affect AMF activity and the degree to which plants rely on fungal-mediated nutrient acquisition (Smith and Smith, 2011; Thirkell et al., 2017; van der Heijden et al., 2015).

Under nutrient-rich conditions, plants frequently reduce carbon allocation to AMF partners, potentially weakening the relationship between EGT accumulation and functional nutrient exchange (Johnson et al., 1997; Walder and van der Heijden, 2015). In contrast, nutrient-limited systems may increase plant dependence on fungal nutrient acquisition and thereby strengthen the sensitivity of EGT accumulation to differences in symbiotic performance among crop varieties.

### EGT and the broader dimensions of AMF functionality

Functional plant–AMF interactions encompass considerably more than nutrient exchange. AMF can influence pathogen suppression, drought and abiotic stress tolerance, hormonal signalling, defence priming, root development, and interactions with other rhizosphere microorganisms (Wehner et al., 2010; Mustafa et al., 2017; Begum et al., 2019; Zhang et al., 2022). Whether EGT accumulation reflects these broader dimensions of AMF functionality, however, remains unknown. Disentangling these relationships will require experiments that quantify EGT alongside independent measures representing different dimensions of AMF functionality. If EGT reflects multiple dimensions of functional plant–AMF interactions, it could provide a particularly useful indicator of symbiotic resilience under climate change, where crops are increasingly exposed to drought, heat, and other environmental extremes.

### Experimental approaches to test the EGT–AMF hypothesis

The conceptual framework proposed here (visualized in **Figure 2**) builds on recent evidence that AMF are involved in EGT accumulation in plants (Beelman et al., 2022; Carrara et al., 2023, 2024). The key question for future research is therefore not simply whether AMF influence plant EGT accumulation, but whether variation in EGT concentration can provide information about the functional performance of plant–AMF interactions, including differences among host genotypes, fungal partners, and environmental conditions. Several experimental approaches could be used to address this question.

**Figure 2:**
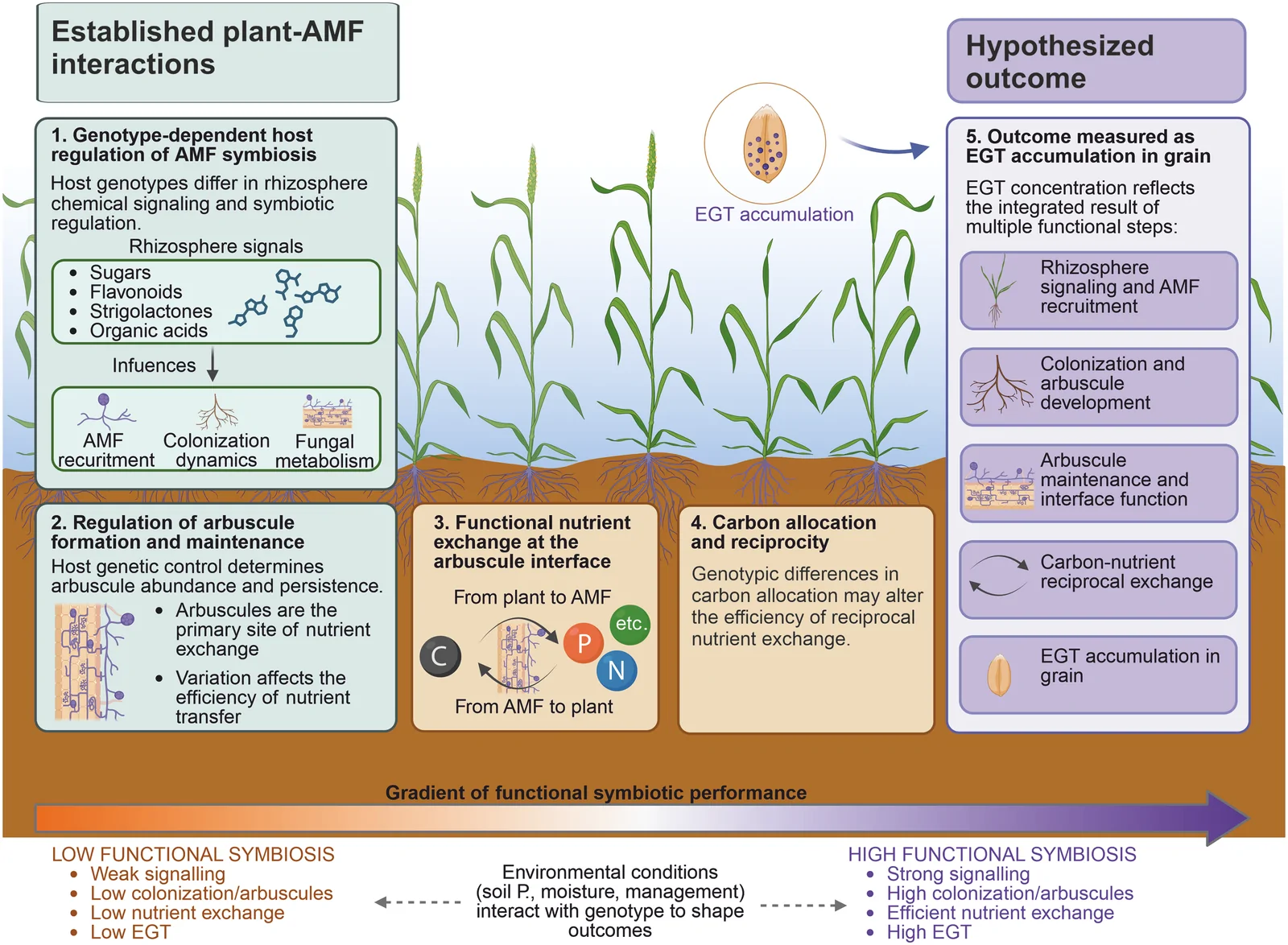
Conceptual framework linking EGT accumulation to functional plant–AMF interactions. Host genotype, rhizosphere signaling, environmental conditions, and AMF community composition collectively influence fungal colonization, arbuscule function, and reciprocal nutrient exchange between plant and fungus. These processes may contribute to variation in plant EGT accumulation together with additional host physiological and transport-related mechanisms independent of AMF functionality. We hypothesize that EGT concentration integrates aspects of functional AMF activity more effectively than root colonization measurements alone and may therefore provide a scalable proxy for assessing plant responsiveness to AMF in low-input agricultural systems. Key references for established mechanisms include Akiyama et al., 2005; Gutjahr and Parniske, 2013; Kiers et al., 2011; Fellbaum et al., 2014 and Thirkell et al., 2020.

First, the dependence of EGT accumulation on AMF-associated processes could be tested by experimentally manipulating AMF presence or functionality. Comparisons between plants grown in sterilized and non-sterilized soils, for example, could determine whether EGT accumulation is reduced when access to native AMF and other soil microorganisms is removed. Where suitable plant material is available, comparisons involving mycorrhiza-defective or mycorrhiza-impaired genotypes would provide a more specific test of the contribution of functional AMF symbiosis. Controlled inoculation experiments could provide a complementary approach by comparing plants with and without defined AMF partners. These experiments would extend existing evidence for an AMF–EGT relationship by testing whether experimentally altering the symbiosis produces corresponding changes in plant EGT accumulation.

Second, if genotype-dependent differences in EGT accumulation reflect variation in host regulation of functional plant–AMF interactions, crop genotypes grown under standardized environmental and microbial conditions should exhibit reproducible differences in EGT concentration that depend on AMF activity. Factorial experiments in which multiple crop genotypes are grown with and without AMF would allow genotype × AMF interactions to be quantified directly.

Third, EGT concentration should be compared with independent measures of AMF-mediated nutrient exchange. Isotope-labeling experiments involving phosphorus or nitrogen transfer in compartmentalized growth systems could quantify the actual contribution of AMF to plant nutrient acquisition. Combining isotopic tracing with EGT measurements would therefore allow the proposed EGT–AMF relationship to be evaluated against a direct functional measure rather than root colonization alone. A positive association between EGT accumulation and isotopically quantified nutrient transfer, particularly across different host genotypes, would provide substantially stronger evidence that EGT reflects functional symbiotic activity.

Fourth, the contribution of fungal identity could be investigated using controlled inoculation experiments with different AMF species or isolates. AMF taxa can differ considerably in their effects on host nutrient acquisition and plant performance, and host responses can likewise depend on the particular plant–fungus combination. If EGT accumulation reflects functional symbiotic performance, variation among AMF partners should therefore be associated with corresponding differences in EGT accumulation and nutrient transfer. Conversely, a lack of correspondence between EGT and independently measured fungal benefits would suggest that EGT is influenced predominantly by other processes.

Fifth, environmental nutrient availability should be considered when evaluating the strength and consistency of the EGT–AMF relationship. Under low-phosphorus conditions, plants may depend more strongly on fungal-mediated nutrient acquisition, potentially increasing the contribution of AMF-associated processes to EGT accumulation. EGT concentrations and AMF-mediated nutrient transfer would therefore be expected to show stronger associations under nutrient-limited than under nutrient-rich conditions.

Together, these approaches would move beyond demonstrating an association between AMF and EGT accumulation and test the more specific hypothesis proposed here: that EGT concentration contains information about how effectively a plant–AMF association functions.

## Conclusions and future directions

Functional interactions between crop plants and arbuscular mycorrhizal fungi are increasingly recognized as important components of sustainable agricultural systems. However, scalable approaches for evaluating the functional performance of plant–AMF interactions remain limited.

Current evidence suggests that EGT accumulation in crop tissues is linked to AMF-associated processes and that crop genotype influence EGT accumulation patterns. We propose that EGT concentration is a promising candidate biochemical indicator of integrated plant–fungal interaction dynamics and that it may be particularly informative in this context because, unlike most nutrients exchanged through AMF symbiosis, it appears to depend largely on microbial production and AMF-associated uptake pathways.

The conceptual framework presented here highlights several mechanisms through which genotype-dependent differences in EGT accumulation could emerge, including variation in symbiotic regulation, nutrient exchange efficiency, rhizosphere microbial interactions, and plant physiological transport processes. At the same time, substantial gaps remain in our understanding of the causal relationship between EGT accumulation and functional AMF-mediated nutrient acquisition.

Further experimental validation integrating EGT quantification with isotopic nutrient tracing, controlled inoculation studies, and plant performance experiments under different fertility regimes, will be necessary to determine the robustness and generality of these relationships. If validated across crop systems and environmental conditions, EGT quantification could provide a scalable tool for investigating crop variation in responsiveness to AMF-associated nutrient exchange.

A better understanding of these relationships may contribute to the development of crop breeding and management strategies better suited for low-input agricultural systems while also improving understanding of how microbial symbioses influence the nutritional quality of food crops.

## Data Availability

The raw data supporting the conclusions of this article will be made available by the authors, without undue reservation.
